# Effect of annealing on a pseudogap state in untwinned YBa_2_Cu_3_O_7−δ_ single crystals

**DOI:** 10.1038/s41598-019-45286-w

**Published:** 2019-06-25

**Authors:** A. L. Solovjov, E. V. Petrenko, L. V. Omelchenko, R. V. Vovk, I. L. Goulatis, A. Chroneos

**Affiliations:** 1 B. I. Verkin Institute for Low Temperature Physics and Engineering of National Academy of Science of Ukraine, 47 Nauki ave., 61103 Kharkov, Ukraine; 2 Physics Department, V. Karazin Kharkiv National University, Svobody Sq. 4, 61077 Kharkiv, Ukraine; 3 Department of Materials, Imperial College, London, SW7 2AZ UK; 4 Faculty of Engineering, Environment and Computing, Coventry University, Priory Street, Coventry, CV1 5FB United Kingdom

**Keywords:** Electronic devices, Superconducting properties and materials

## Abstract

The effect of annealing both in the oxygen atmosphere and at room temperatures on physical properties such as the pseudogap (Δ*(T)) and excess conductivity (σ′(T)) of untwined YBa_2_Cu_3_O_7−δ_ (YBCO) single crystal with a small deviation from oxygen stoichiometry is studied. It was revealed that as the charge carrier density, n_f_, increases, Т_с_ also slightly increases, whereas the temperature of the pseudogap opening, T*, decreases noticeably, which is consistent with the phase diagram (PD) of cuprates. The excess conductivity in the vicinity of T_c_ is represented by the Aslamazov-Larkin and Hikami-Larkin fluctuation theories, illustrating the three-dimensional to two-dimensional (i.e. 3D-2D) crossover with an increase in temperature. The crossover temperature T_0_ determines the coherence length along the *c* axis is ξ_c_(0) = 0.86 Å, that is 2.6 times larger than for optimally doped YBCO single crystals with defects. Taking into account the short coherence length in high-temperature superconductors, in the model of free charge carriers the phase relaxation time of fluctuating Cooper pairs is determined, τ_φ_ (100 K) = (4.55 ± 0.4) · 10^−13^ s, which is slightly (1.2 times) larger than in well-structured YBCO films, and as in films, does not depend on n_f_. It is shown that Δ*(T) at different annealing stages practically does not change its shape. As in the well-structured YBCO films, Δ*(T) demonstrates maximum at T_pair_~124 K which depends weakly on n_f_. However, the maximum value of Δ*(T_pair_) increases with increasing n_f_, as it follows from the PD of cuprates. Comparing the experimental data with the Peters-Bauer theory we estimated the density of local pairs <n_↑_n_↓_> ≈ 0.3 near T_c_ that is a common value for high-temperature superconductors.

## Introduction

The mechanism of superconducting pairing in high-temperature superconductors (HTSCs), which makes it possible to obtain paired fermions at temperatures as higher as ~100 K, remains rather debatable^1–9^. To clarify the issue, the study of superconducting (SC) fluctuations has attracted considerable attention in the research of HTSCs^10–12^ (and references therein). This because it is related to the nature of the pseudogap (PG), which is known to open in underdoped cuprates at a temperature T*, much above the superconducting transition temperature T_c_^2–5,8–11^. Above T* the dc resistivity, ρ(T), of optimally doped (OD) and moderately underdoped cuprates is known to be linear^13^. In the framework of the Nearly Antiferromagnetic Fermi Liquid model^14^ it was proven that this linearity is a specific feature of HTSCs that is represented by the stability of the Fermi surface (FS). Notably, at T < T* not only all the properties of HTSCs measured by various experimental methods^15^ change, but the density of electronic states at the Fermi level begins to decrease^16,17^, which by definition is called a pseudogap^11,18^. It is also assumed that below the PG temperature T* the rearrangement of the FS may begin^2,3,18,19^. Accordingly, at T*, the resistivity curve deviates downward from the linearity leading to excess conductivity σ′(T) as the difference between determined conductivity σ(T) = 1/ρ(T) and extrapolated normal-state conductivity σ_N_(T) = 1/ρ_N_(T)^4,5,11,20,21^.

Importantly, the SC fluctuations are responsible for a relatively short part of the entire excess conductivity, ~15 K above T_c_, which is thus called the fluctuation conductivity (FLC)^10–12,20^ (and references therein). In such a scenario, the long-range coherence is lost at T_c_ due to fluctuations of the phase of the superconducting order parameter^8,22,23^. The FLC region would then be marked by (preformed) fluctuating Cooper pairs (FCPs), which obey the Aslamasov-Larkin (AL)^24^ and Hikami-Larkin (HL) (Maki-Thompson (MT) term)^25^ fluctuation theories, and take the role of the precursor to the SC state^1,8,9^. In the FLC region, the FCPs behave in a good many way like conventional SC pairs without the long-range coherence (known as “short-range phase correlations”)^2,6,8,9^, which have to obey the BCS theory^26^. The question is, what happens with the FCPs, which also called the local pairs (LPs)^11^, with increasing temperature, as there are usually no any particularities on the resistive curve up to T*. In accordance with the theory of systems with reduced charge carrier density^27–30^, along with an increase in T, the in-plane coherence length, ξ_ab_(T), which determines the pair size, decreases. At the same time, the bonding energy of the LPs, ε_b_ ~ 1/ξ_ab_^2 ^^27,30^, noticeably increases. As a result, the LPs should change their properties^9,11,29,30^. Eventually, the FCPs are transformed into so-called strongly bound bosons^9,28–30^, very small but tightly coupled pairs, which obey the Bose-Einstein condensation theory^9,27,30^. Thus, the theory predicts the BCS-BEC transition in HTSCs as T increases, which was observed experimentally^11,12,31^.

Nevertheless, there is still the question of the pairing mechanism, which allows the existence of bound fermions at temperatures significantly higher than T_c_. Obviously, in HTSCs, in addition to the electron–phonon interaction, some other mechanism of interaction, most likely of a magnetic type should act^2–5,31^ (and references therein). As a result, it is proposed to consider both spin-density waves (SDW)^2,3,18^, charge-density waves (CDW)^2–5^ and charge order (CO)^19^ (and references therein) in order to explain the pairing mechanism of HTSCs in the PG state. However, in YBCO, the CO onset temperature appears always to be lower than T*^19^ and the temperature ranges and density of charge carriers indicated in the studies that assume the existence of the SDW, CDW and CO mechanisms differ significantly. As a result, the proposed new phase diagrams of cuprates are also very different^3–5,9,18,19^. Thus, despite the tremendous efforts towards this end there is still no consensus on the physical nature of the PG (refer to^1–11,18,19,28–32^ and references therein).

It is well established now, that all properties of HTSC cuprates are determined by the density of charge carriers, n_f_, which can vary over a wide range upon doping^2–5,10–13,18–21^. In YBa_2_Cu_3_O_7−δ_, n_f_ changes as a result of oxygen intercalation, and the maximum T_c_ ~ 92 K corresponds to a stoichiometric material (i.e. δ = 0)^13^. To transfer YBCO to the so-called overdoped regime, it is necessary to use Ca doping^3^. Usually a set of samples with different n_f_ is used for measurements^10–13,20^. In the manufacture of HTSC films, each n_f_ value is determined by the manufacturing conditions (usually the oxygen pressure in the chamber) of each specific sample^19,33^. As a result, film samples may differ in their structure, number of defects etc. The advantage of single crystals is that their n_f_ can vary noticeably during annealing of the sample in an oxygen atmosphere^13,34^. However, in the case of a strong change in n_f_, various defects may also appear in the samples^21,35^ (cf. the thermodynamic parameters of defect processes in these high T_c_ superconductors have been found to obey the thermodynamical *c*BΩ model -where *c* stands for a dimensionless factor that may be considered in a first approximation to be independent of temperature and pressure, B is the isothermal bulk modulus and Ω the mean volume per atom^36,37^ - in a similar fashion as in the case of the so-called superionic conductors e.g. β-PbF_2_^38^). Therefore, it seems highly desirable to find out how the properties of the same sample, first of all FLC and PG, can change, if somehow the density of charge carriers in the sample varies in a relatively small range.

In the present study we take advantage of single crystals to study FLC and PG in untwined YBCO single crystal with n_f_ close to optimal doping (T_c_ = 91.6 K), when n_f_ changes upon annealing in an oxygen atmosphere. We have studied three samples with different n_f_. For a short notation, we name these samples A1, A2 and A3 (Table 1 and experimental methods). The fluctuation contributions to σ′(*Т*) were derived from the dc resistivity ρ(T) measurements, and temperature dependences of PG, Δ*(T), as a function of n_f_ were calculated. The results show that in the range of SC fluctuation near T_c_, σ′(*Т*) is adequately interpreted by the AL and HL fluctuation theories. It was determined that Δ*(T), as expected, increases upon annealing and is in good agreement with the Peters-Bauer theory (PB)^6^ near (T_c_). The implications of these findings will be discussed.

**Table 1:** Determined parameters.

Sample	ρ(100 K) (µΩcm)	T_c_ (K)	T_c_^mf^ (K)	T_G_ (K)	T_o_ (K)	T_01_ (K)	ΔT_fl_ (K)	d_01_ (Å)	ξ_c_(0) (Å)	C_3D_
А1	47.2	91.6	91.84	91.90	92.34	97.4	5.5	3.50	0.86	1.34
А2	47.0	91.7	91.93	91.98	92.39	96.9	4.9	3.54	0.84	1.25
А3	46.3	91.9	91.98	92.02	92.42	97.3	5.3	3.42	0.81	1.27

## Results and Discussion

### Resistivity

**Figure 1:**
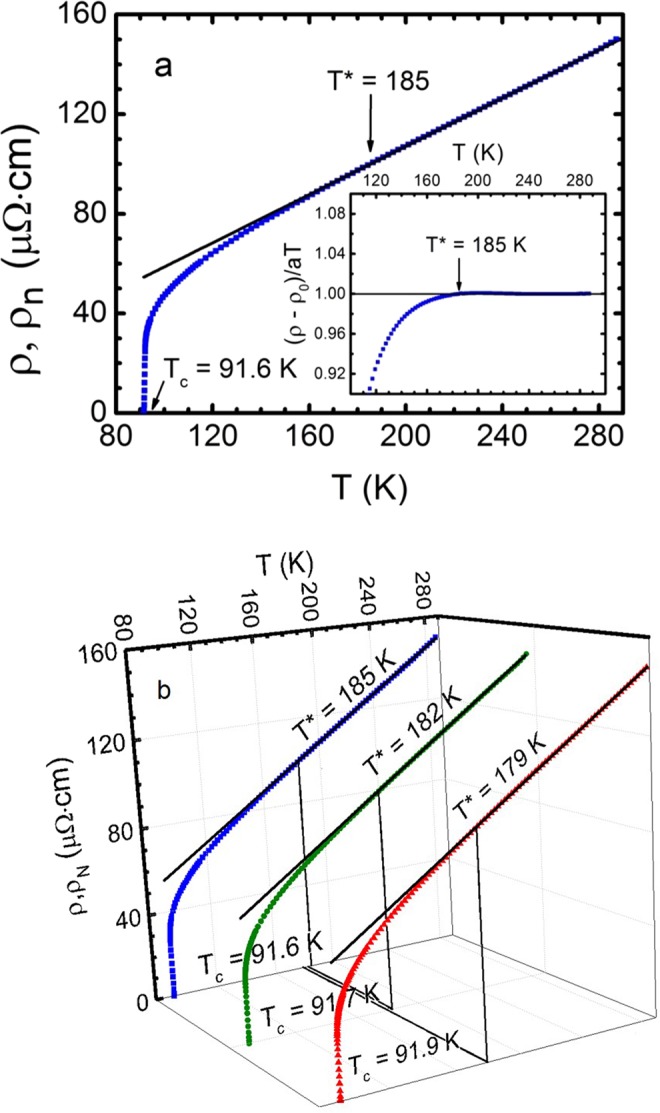
Panel a. Plot of ρ(T) dependence for untwined YBa_2_Cu_3_O_7−δ_ single crystal after annealing in oxygen (sample A1, squares). Insert: The method of determining T*, using criterion [ρ(*T*) − ρ_0_]/*aT* = 1 (sample A1)^39^. Panel b. The plot of ρ(T) of the same single crystal for different annealing stages: A1 (blue squares), A2 (green dots) and A3 (red triangles). The solid lines determine ρ_N_(T), extrapolated to the low-temperature region.

The dependence on temperature of the resistivity (i.e. ρ(T) = ρ_ab_(T)) of untwined YBa_2_Cu_3_O_7−δ_ crystals are shown in Fig. 1. As ρ(T) of the samples differ insignificantly, to simplify the figure on the upper panel (Fig. 1a), only the resistive curve of sample A1 is shown. The corresponding dependences of ρ(T) for all three samples are shown as a three-dimensional (3D) graph in Fig. 1b. For temperatures above T* = (185 ± 0.5) K (A1), (182 ± 0.5) K (A2), (179 ± 0.5) K (A3) and up to 300 K, all the ρ(*T*) dependences are linear and are described by a gradient *a* = *d*ρ/*dT* = 0.484 (A1), 0.488 (A2) and 0.478 (A3) μΩ⋅cm/K, which slightly changes with annealing. The gradient was calculated by approximating the experimentally derived curves and confirmed the linear behaviour of ρ(T) with a mean-root-square error of 0.009 ± 0.002 in the specified temperature range for all samples. As mentioned above, the PG temperature T* ≫ T_c_ was defined as a temperature at which the resistive curve deviates downward from the linearity (Fig. 1). The more precise approach to determine T* with accuracy ± 1 K is to explore the criterion [ρ(*T*) − ρ _0_]*/aT* = 1^39^ (insert in Fig. 1a), where *a* designates the slope of the extrapolated normal-state resistivity, ρ_*N*_(*T*), and ρ_0_ is its intercept with the *y* axis. Both methods give the same T*.

In the process of annealing, with increasing the oxygen content, T_c_ slightly increases, and ρ(T) slightly decreases (Fig. 1b). This is not surprising, since the samples are actually on top of the PD. At the same time, T* decreases much more perceptibly, in full agreement with the PD of cuprates^3–5,13,18,21^ (and Tables 1, 2 and 3). The main difference between the untwined YBCO and single crystal containing defects, presumably in the form of twin boundaries (TB)^21,40^, is much higher T* value. Usually, in optimally doped YBCO single crystals with T_c_ ~ 91.1 K, but containing defects in the form of TB, T* ~ 140 K^41^. It is assumed that the defects prevent the establishment of phase coherence of LPs (paired fermions) and, thus, effectively reduce T*^21,42^. At the same time, in well-structured YBCO films^11,20^, a sample with T_c_ ~ 88 K has T* ~ 200 K, which is much closer to T* = 185 K, observed for the untwined YBCO single crystal A1 with T_c_ = 91.6 K. Therefore, it can be assumed that, by their properties, YBCO single crystals, which do not contain TB, are closer to well-structured films. This conclusion is supported by the analysis of the results of the FLC and PG study.

**Table 2:** Determined parameters.

Sample	R_H_(100 K) (10^−9^m^3^/C)	ρ(100 K)•C_3D_ (µΩcm)	n_f_ (10^21^ cm^−3^)	n_s_ (10^14^ cm^−2^)	μ_H_ (cm^2^/Vs)	*l* (10^−8^ cm)
А1	2.40	62.9	2.60	3.05	38.2	110.0
А2	2.39	58.6	2.62	3.06	40.8	117.8
А3	2.38	58.8	2.63	3.07	40.5	117.1

**Table 3:** Determined parameters.

Sample	ξ_ab_ (Å)	v_F_ (10^7^ cm/s)	m^*^/m_0_	τ(100 K) (10^−13^ s)	τ_φ_(100 K) β (10^−13^ sK)	β	τ_φ_(100 K) (10^−13^ s)
А1	11.00	1.04	4.91	1.06	54.68	12.03	4.55
А2	10.50	1.01	5.04	1.17	59.83	13.49	4.43
А3	10.35	1.04	4.92	1.13	60.73	13.61	4.46

### Fluctuation conductivity

In the resistive measurements, PG is evident as a deviation of the resistivity ρ_*ab*_(*T*) = ρ(*T*), determined in the *ab* plane, from a linear dependence at high temperatures to smaller values (refer to Fig. 1). The result is excess conductivity expressed by σ′(*Т*) = σ(*T*) − σ_*N*_(*T*) = [1/ρ(*T*)] − [1/ρ_*N*_(*T*)], or

1 $$\sigma ^{\prime} ({T})=[{{\rm{\rho }}}_{N}(T)-{\rm{\rho }}(T)]/[{\rm{\rho }}(T){{\rm{\rho }}}_{N}(T)],$$

where ρ_*N*_(*T*) = *aT* + *b* is the sample resistivity in the normal state that is extrapolated to the low temperature region^4,5,20,43^. As mentioned above, according to the model^14^, the linear dependence of ρ(*T*) above T* is the normal state of HTSCs that characterizes by the stability of the FS^2,3,14,19^.

According to recent concepts^4–11,21^, a small value of the coherence length in conjunction with a quasi-layered structure of the HTSCs leads to the formation of a noticeable area of SC fluctuations on the ρ(*T*) in the proximity of T_c_, where σ′(*Т*) follows conventional fluctuation theories^11,20,44–46^. At the same time, changes in oxygen content, the presence of impurities, and/or structural defects have a considerable impact on σ′(*Т*) and, accordingly, on the implementation of various FLC modes above T_c_^20,47–49^.

Fluctuation conductivity for (or of) all the samples was determined by analyzing the excess conductivity, which was calculated in the standard method in accordance with Eq. (1). The FLC analysis was performed within the model of local pairs^1,11,20,21^, in which the presence of paired fermions (LPs) in HTSCs is assumed in the temperature range T_c_ < T < T*^1,6–9,20^. Firstly, the mean-field temperature T_c_^mf^ > T_c_ needs to be determined limiting the region of critical fluctuations near T_c_, where the mean-field theory does not work^26^. In addition, T_c_^mf^ determines the reduced temperature

2 $$\varepsilon =\frac{T-{T}_{c}^{mf}}{{T}_{c}^{mf}}$$

that appears in all equations. From this it is clear that the correct determination of ${T}_{c}^{mf}$ plays a key role in the calculations of both FLC and PG. At the vicinity of T_c_, the coherence length in the *c* axis (ξ_c_(T)) is greater than d. d ≈ 11.7 Å^50^ is the *c* axis lattice parameter of the YBCO unit cell^25,46^. In this case, the FCPs associate throughout the superconductor and form a three-dimensional (3D) state of HTSC^20,25,46^. Therefore, at the proximity of T_c_, the FLC can be described by the 3D equation of the Aslamazov-Larkin (AL) theory^11,24^ with the critical exponent λ = −1/2, which determines the FLC in any 3D system:

3 $${\sigma ^{\prime} }_{AL3D}={C}_{3D}\frac{{e}^{2}}{32{\rm{\hbar }}{{\rm{\xi }}}_{c}(0)}{\varepsilon }^{-\frac{1}{2}}$$

**Figure 2:**
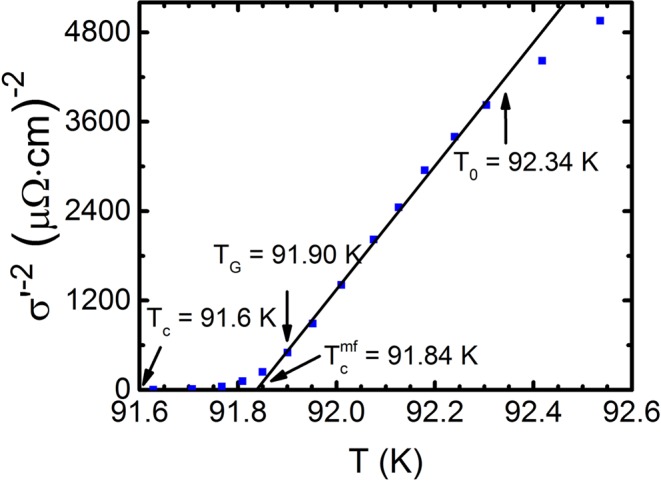
Dependence of σ′^−2^(T) for the untwined YBa_2_Cu_3_O_7-δ_ single crystal after annealing in oxygen (sample A1). Arrows indicate Т_с_, T_с_^mf^, the Ginzburg temperature T_G_ and 3D-2D crossover temperature T_0_. A straight line is to guide the eye.

**Figure 3:**
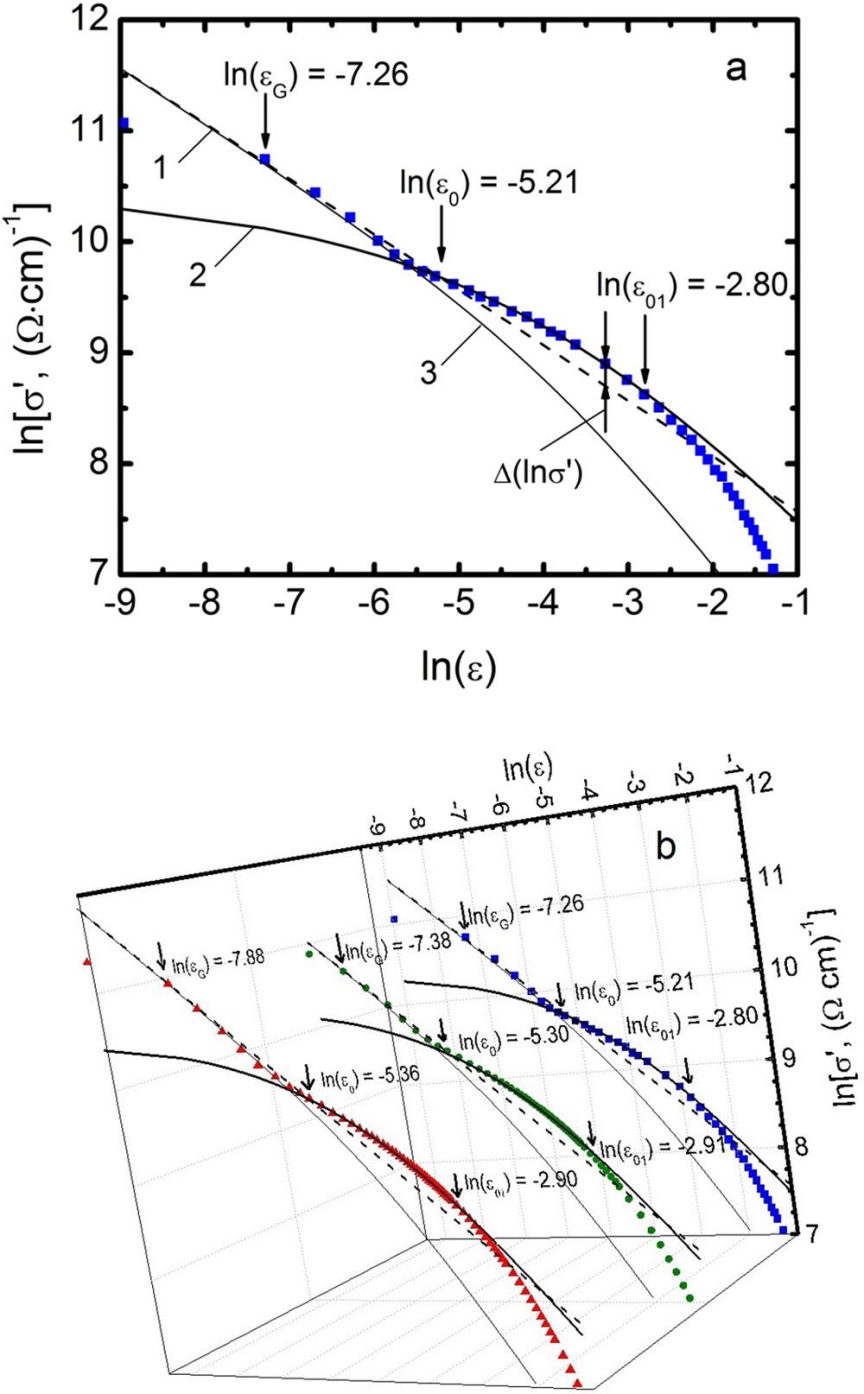
Panel a. lnσ′ vs lnε for the sample A1 (squares) in comparison with fluctuation theories: 3D AL (dashed line (1)), 2D MT (solid curve (2)) and LD (solid thin curve (3)). The T_01_ (lnε_01_) determines the range of the SC fluctuations, T_0_ (lnε_0_) is the temperature of the 3D-2D crossover and T_G_ (lnε_G_) is the Ginzburg temperature. Panel b: The same dependencies for all three samples: A1 - blue squares, A2 - green dots and A3 - red triangles.

Here σ′(Т) ~ ε^−1/2^. It can be shown that σ′^−2^(Т) ~ ε~ T − T_c_^mf^ and vanishes at T = T_c_^mf^ (refer to Fig. 2), which enables the determination of both T_c_^mf^ and ε with high accuracy^11,20,51^. Also in Fig. 2, the arrows show T_c_, the Ginzburg temperature T_G_, down to which the fluctuation theories are valid^46,47^, and the T of the 3D-2D crossover T_0_ limiting the area of 3D fluctuations. Notably, above T_0_ = 92.34 K (refer to Fig. 2), the data deviate to the right from the linear dependence, which indicates the presence of 2D Maki-Thompson (MT) contribution to the FLC^25,46^. Having determined ε, we construct the dependence lnσ′(lnε) (Fig. 3). Figure 3a shows the corresponding dependence for the base sample A1. Expectedly at the vicinity of T_c_, in the interval Т_G_ — T_0_ (ln(ε_0_) = −5.21), the FLC is well modelled by the AL fluctuation contribution (3) for the 3D system. In double logarithmic coordinates this is the dashed line (1) with slope λ = −1/2. As mentioned above, it implies that the classical three-dimensional FLC materializes in HTSC for *Т → Т*_*с*_ and ξ_*с*_(*Т*) > *d*^11,20,43^. Above the crossover temperature Т_0_ ξ_*с*_(*Т*) < *d*^11,21,25,46^, and this is no longer a 3D regime. However, as before, ξ_*с*_(*Т*) > *d*_01_, where d_01_ ≈ 3.5 Å is the separation of the conducting planes of CuO_2_ in YBCO^50^. Thus, up to temperature Т_01_ (ln(ε_01_) = −2.8, Fig. 3) ξ_*с*_(*Т*) connects the inner planes of CuO_2_ by means of the Josephson interaction^11,20,46^. This is the 2D FLC regime, which is perfectly approximated by the Hikami-Larkin theory 2D-MT equation for HTSCs^25^:

4 $${\sigma }_{MT2D}^{^{\prime} }=\frac{{e}^{2}}{8d{\rm{\hbar }}}\frac{1}{1-\alpha /\delta }{\rm{In}}[(\delta /\alpha )\,\frac{1+\alpha +\sqrt{1+2\alpha }}{1+\delta +\sqrt{1+2\delta }}]{\varepsilon }^{-1}$$

Above Т_01_, the experimental points deviate downward from the theory (Fig. 3) implying that the classical fluctuation theories are not valid. Thus, Т_01_ limits the region of SC fluctuations from above: ΔT_fl_ = T_01_ − T_G_. Conversely, T_G_ limits the region of SC fluctuations from below. As a result, below T_G_, designated as ln(ε_G_) in (Fig. 3a,b), the experimental points also deviate downward from the theory (Fig. 3), suggesting the transition to the range of critical fluctuations or fluctuations of the SC order parameter Δ just near T_c_, where Δ < kT^11,26^.

The thin curves (3) in the figure are constructed according to the Lawrence-Doniach equation (LD)^44^:

5 $${\sigma }_{LD}^{^{\prime} }={C}_{LD}\frac{{e}^{2}}{16{\rm{\hbar }}d\sqrt{1+2\alpha \,}}{\varepsilon }^{-1}$$

The LD model works in case of defects in samples^21,42,51^. In our case, curves (3) lie far from the experimental points, which confirm the absence of defects (primarily TB) in our samples.

Notably, in this case the maximum distance between the MT curve (2) and the extrapolated AL straight line (1), Δlnσ′~0.1, which is typical for nonmagnetic YBCO^11,20^. In magnetic superconductors, such as SmFeAsO_0.85_, the MT curve (2) always passes much higher than the extrapolated AL straight line (1)^11^, and in this case Δlnσ′~0.8^31^. Such behavior indicates the presence of a magnetic interaction in HTSCs, which is clearly absent in our non-magnetic untwined single crystal.

At T_0_, ξ_*с*_(*Т*_0_) = *d* = 11.7 Å, which allows us to determine ξ_*с*_(0)^20,21,25,46^

6 $${\xi }_{c}(0)={\rm{d}}\sqrt{{\varepsilon }_{0}}$$

Taking into account that ln(ε_0_) = −5.21 (Fig. 3) and using Eq. (6), we get ξ_*с*_(0) = (0.86 ± 0.02) Å (A1), which is almost 2.6 times the coherence length alongside the c axis obtained for OD YBCO with TB (T_c_ = 91.07 K)^41^. This is most likely due to the fact that in single crystals with defects the region of SC fluctuations is ΔT_fl_ = T_01_ − T_G_ ~ 1.5 K, that is extremely small. While for A1, ΔT_fl_ = T_01_ − T_G_ = 97.4 K − 91.9 K = 5.5 K, that is, 3.7 times more. The result again underlines a noticeable difference in the behavior of YBCO single crystals with and without defects. Additionally, ξ_с_(T_01_) = *d*_01_, and, since ξ_*с*_(0) has already been defined by Eq. (6), we can calculate d_01_ from the relation ξ_с_(0) = *d√ε*_0_ = *d*_01_*√ε*_01_. For sample A1, calculations give d_01_ = (3.5 ± 0.2) Å, in good agreement with the results of structural studies^50^.

In Fig. 3b the dependences of lnσ′(lnε) are shown for all three samples A1–A3. It is seen that with increasing T_c_ all characteristic temperatures also vary slightly. The increase in the absolute value of lnε_0_ results in decrease in ξ_*с*_(0) from 0.86 Å (А1) to 0.81 Å (А3) (Table 1), in full agreement with the theory of superconductivity, where ξ~1/T_c_^26^.

In the above equations

7 $$\alpha =2{(\frac{{\xi }_{c}(0)}{d})}^{2}{\varepsilon }^{-1}$$

is the coupling parameter;

8 $$\delta =\beta \frac{16}{\pi {\rm{\hbar }}}{(\frac{{\xi }_{c}(0)}{d})}^{2}{k}_{B}T{\tau }_{\phi }{\varepsilon }^{-1}$$

is the pair-breaking parameter, and the phase relaxation time τ_φ_ is determined by the equation

9 $${\tau }_{\phi }\beta T=\pi {\rm{\hbar }}/8{k}_{B}{\rm{\varepsilon }}=\,A/\varepsilon $$

where А = 2.998·10^−12^ s K. Here the factor β = 1.203(*l*/ξ_ab_) with *l* being the mean free path and ξ_ab_ the coherence length along the *ab* plane, takes into account the approximation of the clean limit (*l* > ξ) that consistently occurs in HTSCs due to the smallness of ξ(T)^25,44–46^.

### Comparative analysis of phase relaxation time

Having determined the parameters of the FLC analysis, it seems interesting to examine the physical meaning of the short coherence length ξ_ab_(0) in the framework of the simple two-dimensional model of free charge carriers^52–54^. This approach allows us to define a set of additional important parameters of the samples, including τ_φ_, which is actually the lifetime of the FCPs in the range of SC fluctuation. In HTSCs all parameters, including τ_φ_ and Hall coefficient R_H_, are functions of temperature. Consequently, the corresponding calculations, including those in YBCO, are performed at T = 100 K, as is customary in the literature^20,52–54^.

From the FLC analysis, using Eq. (7), we find the coupling parameter α, and then the pair-breaking parameter δ of Eq. (8), which is always ~ 2^20,54^, if all other parameters are correctly determined. Next, we calculate the parameter τ_φ_βТ (refer to Table 3), assuming in Eq. (9) ε = ε_0_^20^. Since it is assumed that T = 100 K, in order to find τ_φ_(100 K), it is necessary to determine the coefficient β = 1.203(*l*/ξ_ab_). For this it is necessary to know the mean free path *l*, which is determined by the density of charge carriers n_f_, and the coherence length in the *ab* plane (i.e. ξ_ab_). The charge carrier density n_f_ can be calculated from the values of the Hall coefficient R_H_, namely n_f_ = r [l/(e R_H_)]^20,54,55^. Here e is the electron charge, and the coefficient r = <τ^2^>/<τ>^2^, where τ is the average time between collisions of charge carriers, actually determines the scattering mechanism in the normal state^55^. Since the scattering mechanism of normal charge carriers in HTSCs, especially in the PG state, is still rather uncertain^56–59^, we assume r = 1.

From the literature for optimally doped YBCO single crystals^3,60^ and YBCO films with a close value of T_c_^20^, we find: R_H_(100 K) ≈ 2.4·10^−9^ m^3^/C for sample A1, and, accordingly, n_f_ = 2.6·10^21^ см^−3^ (Table 2). Continuing the analysis of sample A1, for the carrier density in the planes we obtain n_s_ = n_f_d = 3.05·10^14^ см^−3^. Using the corrected value ρ(100 K) = ρ(100 K)·C_3D_ = 62.9 μΩcm^52,53^, we have μ_H_ = r/(ρne) = 38.2 сm^2^/Vs for the mobility of the Hall carriers. Interestingly, found values of μ_H_ (Table 2) are in good agreement with those obtained in ref.^60^ for YBaCuO untwinned single crystals. It was also shown^60^ that the results of Hall-effect measurements are nor affected by the conduction of the Cu-O chains and the in-plane anisotropy of the CuO_2_ planes. Now, using the formula *l* = (ħμ/e)(2πn_s_)^1/2^, we find the mean free path of the charge carriers in A1: *l* = *υ*_F_τ ≈ 110 Ǻ, where *υ*_F_ is the Fermi velocity. To continue the analysis, the mean value ξ_ab_(0) = 11.0 Ǻ was chosen for A1 from the literature^51–53^.

In the general theory of superconductivity^26^

10 $${\xi }_{0}\sim {\rm{\hbar }}{{\upsilon }}_{{\rm{F}}}/[\pi {\rm{\Delta }}(0)]$$

where Δ(0) is the SC order parameter at T = 0 K. Taking into account that in YBCO Δ(0)/k_B_T_c_ = 5^54,61,62^ and setting ξ_0_ = ξ_ab_(0), for the Fermi velocity we obtain *υ*_F_ = 1.04·10^7^ cm/s, and for the effective mass of charge carriers m*/m_0_ = (ρ*l*)(n_f_e^2^)/(*υ*_F_m_0_) = 4.91 (Table 3). After this, the transport time of the normal carriers τ(100 K) = *l*/*υ*_F_ = 1.06·10^−13^ s can also be calculated. All estimates obtained are in good agreement with the similar results reported for optimally doped YBCO^20,52–54,58,59^.

Finally, we find β (100 K) = [1.203(*l*/ξ_ab_)] = 12.03. Now, using found τ_φ_ (100 K)β = 54.68·10^−13^ sK, we get the value of τ_φ_ (100 K) = 4.55·10^−13^ s. Performing similar calculations for samples A2 and A3 and taking into account the corresponding changes of R_H_ and ξ_ab_(0) with increasing n_f_ (Tables 2 and 3), we obtain τ_φ_ (100 K) = 4.43·10^−13^ s and τ_φ_ (100 K) = 4.46·10^−13^ s (Table 3) in good agreement with similar results obtained by measuring the magnetoresistance on YBCO-PrBCO superlattices^63^ and FLC on YBCO films^20^.

Nevertheless, the mean free path *l* and the Hall mobility μ_H_ are about 2 times, and τ_φ_(100 K) is ~ 1.2 times more than in OD YBCO films^20^, which is most likely a specific property of untwined single crystals^60^. At the same time τ_φ_ (100 K)/τ (100 K) ~ 4 ± 0.2, in excellent agreement with the theory^45^, which takes into account the clean-limit approximation (*l* > ξ), as mentioned above. It should be emphasized that, as in well-structured YBCO films with different n_f_^20^, in the untwined single crystals τ_φ_(100 K) is practically independent on n_f_. This result, apparently, can be considered as a common property of cuprates, at least of compounds based on YBCO.

### Pseudogap analysis

In resistive measurements of cuprates the pseudogap is a deviation at T ≤ T* of the longitudinal resistivity ρ(T) from linearity in the normal phase^11,14,64^. This results to the realization of excess conductivity σ′(T) (refer to Eq. (1)). It is established that if there were no processes in HTSC leading to the opening of the PG at T*, then ρ(T) would preserve its linearity up to T ~ T_c_^4,11,14,27–30^. It is obvious that σ′(T) is a consequence of the PG opening and should enclose details about the magnitude and temperature dependence of the PG^11,23^. Conventional fluctuation theories, modified by Hikami-Larkin^25^ for HTSCs perfectly describe the experimental σ′(T) but only to about ~110 K^10,11,31^. For complete information about the pseudogap, an equation is needed that would describe the experimental curve in all temperature range from T* to T_c_ and would contain PG explicitly. Such an equation was proposed earlier^12^:

11 $$\sigma ^{\prime} (\varepsilon )=\frac{{e}^{2}{A}_{4}[1-\frac{T}{{T}^{\ast }}][exp(-\frac{{{\rm{\Delta }}}^{\ast }}{T})]}{16{\rm{\hbar }}{\xi }_{c}(0)\sqrt{2{\varepsilon }_{c0\,}^{\ast }\,\sinh (2\varepsilon /{\varepsilon }_{c0}^{\ast }})}$$

where (1 − T/T*) takes into consideration the number of LPs formed at T ≤ T*, and (exp(−Δ^*^/T)) determines the dynamics of LPs destruction as T approaches T_c_.

**Figure 4:**
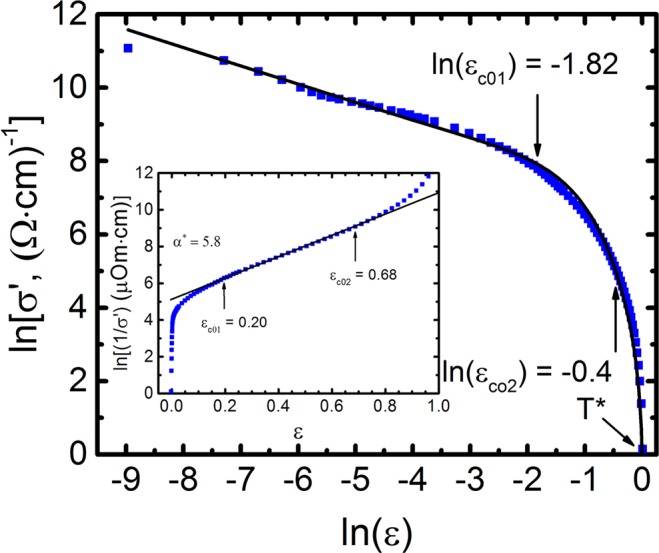
lnσ′ vs lnε (squares) for the untwined YBa_2_Cu_3_O_7–δ_ single crystal (sample A1) in the whole temperature range from T* to T_G_. The solid curve is fit to the data with equation (11) with the set of parameters given in the text. ln(ε_c01_) and ln(ε_c02_) designate the interval of exponential dependence of σ^−1^(ε). In the inset: ln(1/σ′) with respect to ε^65^. Solid line indicates the linear part of the curve between ε_*c*01_ = 0.20 and ε_*c*02_ = 0.68. Corresponding ln(ε_c01_) = −1.82 and ln(ε_*c*02_) = −0.4 are marked by arrows in the main panel. α* = 5.8 is used to determine the parameter ε*_c0_ = 1/α* = 0.17.

**Figure 5:**
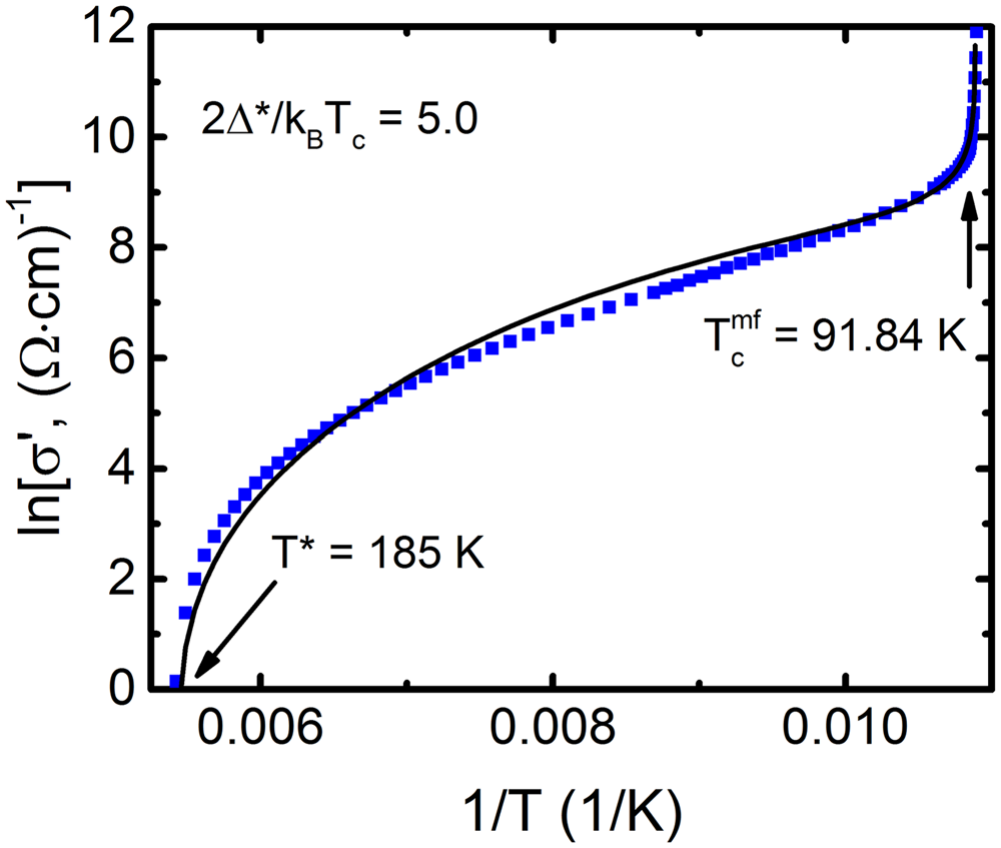
lnσ′ as a function of 1/T (squares) for the sample A1 in the interval from T* to T_c_^mf^. The solid curve is fit to the data with equation (11) with a set of parameters given in Tables 2, 3 and 4. The best fit is obtained when Eq. (11) is calculated with ∆*(*T*_*G*_) = 229 K, that is D* = 2∆*(*T*_*G*_)*/k*_*B*_*T*_*c*_ = 5.0.

Additionally to T*, ε and ξ_c_(0), already defined above, Eq. 11 includes the numerical coefficient A_4_, which is equivalent to the C-factor in the FLC theory^20,51–53^, the theoretical parameter ε_c0_*^65^ and Δ* = Δ*(T_G_). Here it is presumed that Δ*(T_G_) = Δ(0)^66,67^ with Δ being the order parameter of the sample in the SC state, as mentioned above. Importantly, all these parameters can be easily determined within the LP model^11,12,20,31^. We consider this for the case of A1 (refer to Figs 4, 5). In the region lnε_c01_ < lnε < lnε_c02_ or, respectively, ε_c01_ < ε < ε_c02_ (113 K < T < 155 K), σ′^−1^ ~ exp(ε)^65^. This feature turned out to be one of the main properties of most HTSCs^11,31,54^. As a result, in the specified temperature range, ln (σ′^−1^) depends linearly with respect to ε with a slope α* = 5.8 (insert to Fig. 4a), which determines the parameter ε_c0_* = 1/α* = 0.172^65^. This allows the determination of reliable values of ε_c0_* for all samples, which, as established previously^12,31^, significantly impacts the dependence of the theoretical curves shown in Figs 4 and 5, at high T.

**Figure 6:**
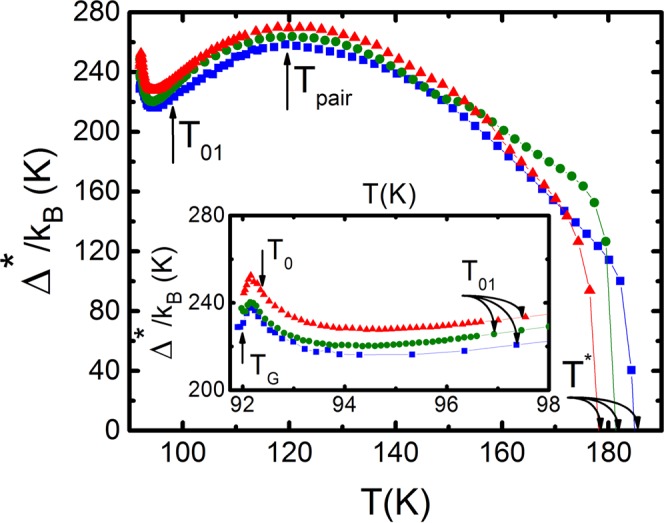
Temperature dependences of Δ*(T), of the untwined YBa_2_Cu_3_O_7−δ_, single crystal, calculated by Eq. (12) for all annealing stages: A1 - blue squares, A2 - green points, A3 - red triangles. Inset: The same dependence for the temperature interval T_G_ < T < T_01_. The arrows show all characteristic temperatures. Solid lines are to guide the eye.

To determine A_4_, we approximate the experiment by the dependence σ′(ε) calculated by Eq. (11), in the vicinity of 3D AL-fluctuations near T_c_ (refer to Fig. 4). lnσ′(lnε) is in essence a linear dependence of ε (i.e. the reduced temperature) and has a slope λ = −1/2. To find Δ*(T_G_) used in Eq. (11), we construct the curve lnσ′(1/T) using all the parameters found^68^ (refer to Fig. 5). Here, the gradient of the theoretical curve (11) is highly influenced by the value of Δ*(T_G_)^12,20,31^. The best approximation is achieved when the Bardeen-Cooper-Schrieffer (BCS) ratio D* = 2Δ*(T_G_)/k_B_T_c_ is 5.0 ± 0.1, which corresponds to the strong-coupling limit characteristic for YBCO. Accordingly, we obtain: Δ*(T_G_)/k_B_ ≈ 229 K in good agreement with the experimental value Δ*(T_G_)/k_B_ ≈ 228 K (see Fig. 6). Similar results were obtained for samples A2 and A3 (refer to Table 4).

**Table 4:** Experimental Parameters.

Sample	T^*^ (K)	α*	ε^*^_с0_	A_4_	∆*(T_G_) (K)	∆*_max_(T_pair_) (K)	T_pair_ (K)	D^*^ (K)
A1	185	5.8	0.17	33	229	258	119.3	5.0
A2	182	5.9	0.17	33	237	264	120.6	5.1
A3	179	5.8	0.17	37	244	270	120.7	5.3

Solving equation (11) for the PG, Δ*(T), we obtain^11,12^ over the entire temperature range from T* to T_c_^mf^

12 $${{\rm{\Delta }}}^{\ast }({\rm{T}})=T\,{\rm{In}}\frac{{e}^{2}{A}_{4}(1-\frac{T}{{T}^{\ast }})}{\sigma ^{\prime} (T)16{\rm{\hbar }}{\xi }_{c}(0)\sqrt{2{\varepsilon }_{c0\,}^{\ast }\,\sinh (2\varepsilon /{\varepsilon }_{c0}^{\ast }})}$$

Here σ′(T) is the experimentally determined excess conductivity and the remaining parameters are already defined within the LP model (Tables 1 and 4). The fact that σ′(T) is given by Eq. (11) (refer to Fig. 4) suggests that Eq. (12) gives reliable values of both the magnitude and the temperature dependence of Δ*(T). The dependence Δ*(T) for sample A1, constructed by the formula (12), using the following parameters extracted from the experiment: T_c_^mf^ = 91.84 K, T* = 185 K, ξ_c_(0) = 0.86 Å, ε_c0_* = 0.17, A_4_ = 33, Δ*(T_G_)/k_B_ = 229 K, shown in Fig. 6. Also shown are the dependencies Δ*(T) for samples A2 and A3, calculated in a similar way with the corresponding set of parameters given in Tables 1 and 4.

All the curves in Fig. 6 have the shape typical for YBCO films, with a maximum at Т = T_pair_ ≈ 124 K, which is close to T_pair_ ≈ 130 K usually observed in well-structured YBCO films^12,54^, and a minimum at Т ≈ Т_01_^31,41^. It can be seen that, in full accordance with the phase diagram of cuprates, Δ*_max_(Т_pair_)/k_B_ expectedly increases from 258 K (A1) to 270 K (A3) along with an increase in n_f_. The BCS ratio D* = 2Δ*(T_G_)/k_B_T_c_ also increases from 5.0 to 5.3. At the same time, T_pair_ practically does not change (Table 4), which is reasonable, given the high T_c_ of the samples. As mentioned above, according to the theory of systems with low n_f_^8,9,27–30^, above T_pair_, LPs must exist in the form of strongly bound bosons, which obey BEC. Below T_pair_ the LPs must be converted to FCPs, which are subject to BCS theory. Thus, T_pair_ separates both regimes^8–12,28–30^. The minimum Δ*(T) at T ≈ Т_01_ is also observed on all HTSCs, including pnictides^11^ and single crystals of FeSe^69^. Accordingly, when approaching to T_c_, the maximum Δ*(T) always occurs just below T_0_, and the minimum at T = T_G_^31,41^ (inset in Fig. 6). Below T_G_, there is an abrupt jump Δ*(T) at Т → T_c_^mf^, however, this is already a transition to the region of critical fluctuations, where the LP model does not work. Thus, the approach in the framework of the LP model makes it possible to determine the exact values of T_G_ and, as a consequence, to obtain reliable values of Δ*(T_G_) ≈ Δ*(T_c_) ≈ Δ(0)^12,54,65–67^ (Table 4).

**Figure 7:**
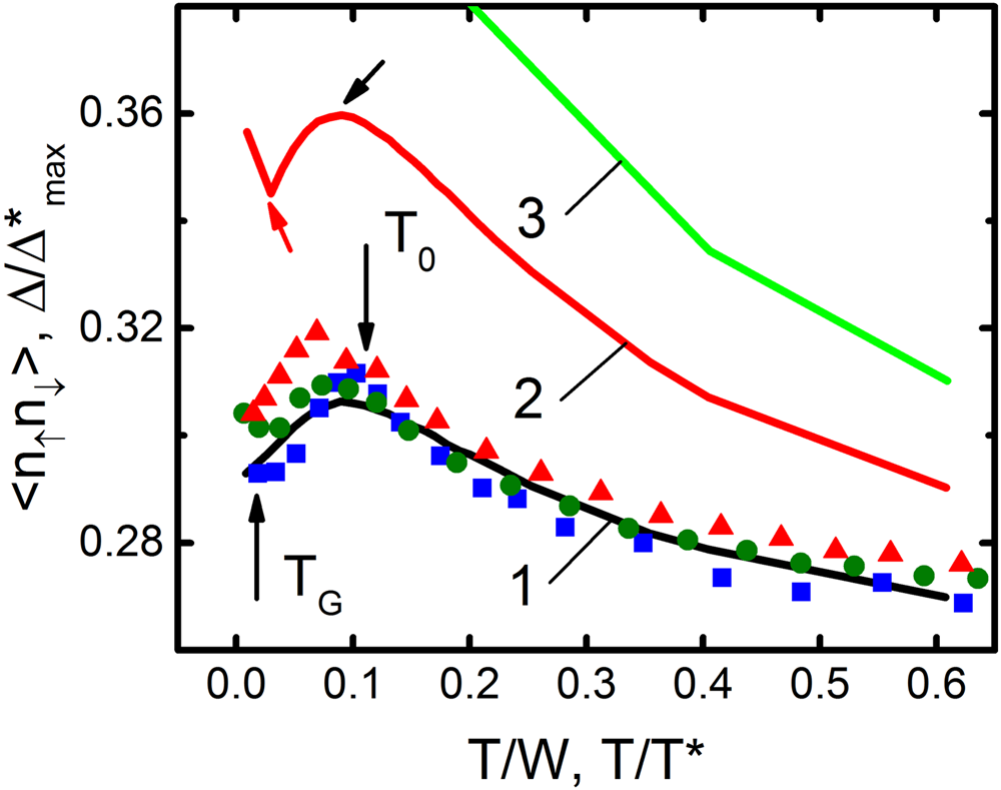
Solid curves display the density of local pairs <n_↑_n_↓_> as a function of temperature for different values of the interaction energy: U/W = 0.2 (curve 1), U/W  = 0.4 (curve 2) and U/W = 0.6 (curve 3)^6^. The symbols are experimental data for A1 (blue squares), A2 (green points), and A3 (red triangles), after corresponding renormalizations of the dependence Δ*(T) in the interval T_G_ < T < T_01_ (see Text).

It is noteworthy that the shape of the Δ*(T) curves near T_c_, shown in the inset to Fig. 6, is very similar to the temperature dependence of the density of local pairs in HTSCs, <n_↑_n_↓_>, calculated within the three-dimensional attractive Hubbard model for different temperatures, interactions U > 0, and filling factors (the Peters-Bauer model (PB)^6^). Besides, in the calculations the hopping t and the bandwidth W = 12t were used as energy scales. Taking into account the fact that Eq. (12) contains information on the density of local pairs, we tried to compare our data with the results of the PB theory ref.^6^. Having normalized the temperature and PG, respectively, by T*(T) and Δ*_max_, and having adjusted the parameters, we obtained good agreement with the PB theory (refer to Fig. 7). In the process of fitting, Δ*(T_G_)/Δ*_max_ was coincided with the minimum value <n_↑_n_↓_> at the lowest T, and Δ*(T_0_)/Δ*_max_ with the maximum value. Both temperatures in Fig. 7 are indicated by arrows. On the theoretical curve 2, these temperatures are indicated by inclined arrows. Importantly, the same fitting factors were used for all samples.

After fitting, good agreement was found between the experimental Δ*(T)/Δ*_max_ and the theory of PB with the lowest interaction parameter U/W = 0.2, which corresponds to the density of local pairs <n_↑_n_↓_> ≈ 0.3 near T_c_ (Fig. 7). As in the PB theory, the density of LPs in our samples increases (from <n_↑_n_↓_> (T_G_) ≈ 0.292 (A1) to <n_↑_n_↓_> (T_G_) ≈ 0.305 (A3)) with an increase in the interaction energy, which corresponds to an increase in the BCS ratio D* in our case. As the temperature increases both <n_↑_n_↓_> and our data, as expected, decrease (refer to Fig. 7), which seems reasonable. Indeed, the number of FCPs should decrease along with T^3–6,11^. Importantly, at U/W = 0.2 the experimental data is consistent with theory in a wide temperature range, actually in the whole range of SC fluctuations. Whereas, if we compare the data with the theory for larger values of U/W (curves 2 and 3), the data will deviate from the theory already at T/W ~ 0.2. Notably, <n_↑_n_↓_> ≈ 0.3 was also obtained for FeSe single crystals near T_c_^69^. Apparently, such a density of LPs near T_c_ is typical of all HTSCs.

### Conclusions

Using the LP model, we have studied the effect of annealing on the temperature dependences of FLC and PG in untwined OD YBa_2_Cu_3_O_7−δ_ (YBCO) single crystal with a slight increase in the oxygen index (7-δ). The increase in oxygen content and, respectively, n_f_ was carried out by annealing the single crystal both in an oxygen atmosphere (sample A1) and by exposure at room temperature (samples A2 and A3). It is found that with increasing n_f_ in the sample, T_c_ increases, and resistivity decreases. As expected, the increase in T_c_ is rather small, since n_f_ actually corresponds to the maximum of the phase diagram (PD). At the same time, T* decreases more significantly (from 185 K to 179 K), which fully corresponds to PD of the cuprates. The first difference from optimally doped YBCO single crystals containing defects in the form of TB, where T* ~ 140 K^41^, is quite large T* = 185 K (A1). It is assumed that defects interfere with the establishment of phase coherence of LPs (paired fermions) and, thus, effectively reduce T*^21,42^. Importantly, in well-structured YBCO films, the sample with T_c_ ~ 88 K has T* ~ 200 K, which is much closer to T* = 185 K, observed for A1. This result suggests the conclusion that the investigated properties of untwined YBCO single crystals are noticeably closer to the well-structured films, which was confirmed by the results of analysis of both FLC and PG.

The present study demonstrated that in the range of SC fluctuations near T_c_ FLC is consistent with the fluctuation theories of Aslamazov-Larkin (3D term) and Hikami-Larkin (2D-MT term), and demonstrate a 3D-2D crossover when the temperature is increased. T_0_ determines ξ_c_(0) = 0.86 Å (A1), which is 2.6 times higher than in optimally doped defective YBCO single crystals. This is most likely due to the fact that the range of FLC is very small: ΔT_fl_ = T_01_− T_G_ = 97.4 K− 91.9 K = 5.5 K (А1), which, however, is 3.7 times more than in single crystals containing defects, where ΔT_fl_ ~ 1.5 K^41^. According to the theory^8,9^, in the range of SC fluctuations, cuprates retain the finite value of superfluid density n_s_ (T), and the FCPs behave mainly as SC Cooper pairs, but without long-range order (known as “short-range phase correlations”) that is confirmed by a number of experiments^66,70,71^. This result once again underlines a noticeable difference in the behavior of YBCO single crystals with and without defects.

T_01_ also determines d_01_ (distance between the conducting CuO_2_ planes). In this case, regardless of the density of charge carriers, d_01_ ~ 3.5 Å, in agreement with the determinations of structural studies^50^. This result, together with the presence of the fluctuation contribution of 2D-MT in FLC, confirms the good structure of the samples. It should be also noted that with increasing Т_с_, ξ_*с*_(0), as it was found, decreases from 0.86 Å (A1) to 0.81 Å (A3) (Table 1), that is ξ~1/T_c_. The result is fully consistent with the theory of superconductivity, where ξ~ ћ*v*_F_/πk_B_T_c_^26^, because, as well as in the well- structured YBCO films^20^, *v*_F_ is almost independent on n_f_ (Table 3).

Having determined the parameters of the FLC analysis, the physical meaning of the short coherence length ξ_ab_(0) in HTSCs was examined in the framework of the simple two-dimensional model of free charge carriers^52–54^. This approach allowed us to define a set of additional important parameters of the samples, including τ_φ_, which is actually the FCPs lifetime in the range SC fluctuations. Most of the calculated parameters are in good agreement with similar data obtained for OD YBCO^20,52–54,58,59^. It is shown that found τ_φ_(100 K) = (4.49 ± 0.06) 10^−13^ s (Table 3) is only slightly (~1.2 times) more than in well-structured YBCO films, but, as in films, in fact does not depend on n_f_. Accordingly, τ_φ_(100 K)/τ(100 K) ~ 4 is in excellent agreement with the Bieri-Maki-Thompson theory, which takes into account the approximation of the clean limit (*l* > ξ)^41^, which always takes place in HTSCs due to the small value of ξ(T). A certain role in this may be played by the presence of structural and kinematic anisotropy in the system^72–75^.

The PG analysis has shown that the Δ*(Т) curves (Fig. 6) have the shape characteristic of YBCO films^12,54^, with a clear maximum at Т = T_pair_ ≈ 124 K and a minimum at Т ≈ Т_01_^31,41^. According to the theory of systems with low n_f_^8,9,27–30^, T_pair_ separates both BEC and BCS regimes of LPs formation^8–12,27–29^. In full accordance with the PD of cuprates, Δ*_max_(Т_pair_)/k_B_ expectedly increases from 258 K (A1) to 270 K (A3) along with an increase in n_f_ and T_c_ (Fig. 6). The BCS ratio D* = 2Δ^*^(T_G_)/k_B_T_c_ also increases from 5.0 to 5.3, suggesting the expected increase in bonding energy of the LPs^11,27,30^. At the same time, T_pair_ practically does not change (Table 3), which is understandable due to the high T_c_ of the samples. When approaching T_c_, the PG curves show behavior being typical for all HTSCs with a maximum Δ*(T) just below T_0_ and a minimum value at T = T_G_ (inset in Fig. 6). Thus, the approach within the LP model makes it possible to determine the exact values of T_G_ and, as a consequence, to obtain reliable values of Δ*(T_G_)≈ Δ(0)^12,54,65–67^ (Table 4).

Finally, the shape of the Δ*(T) curves near T_c_ (Fig. 6), was found to be very similar to the temperature dependence of the density of LPs in HTSCs <n_↑_n_↓_> calculated within the three-dimensional attractive Hubbard model for different values of temperature, interaction, and filling factors (the Peters-Bauer model (PB)^6^). For the first time, an estimation of the density of local pairs <n_↑_n_↓_> in the optimally doped YBCO was carried out by comparing the experimental data of Δ*(T) with the PB theory (Fig. 7). It was determined that <n_↑_n_↓_> ≈ 0.3 near T_c_, which, likely, is a typical value for HTSCs.

### Experimental methods

The YBa_2_Cu_3_O_7−δ_ single crystals were grown by the solution-melt technology in a gold crucible, according to the procedure described in^76,77^. As is well known, with an increase in the oxygen content a tetra-ortho structural transition occurs in YBa_2_Cu_3_O_7−δ_^78^, which leads to a twinning of the single crystal and the creation of twin boundaries (TB), minimizing its elastic energy^76^. To obtain an untwined sample, the crystal was untwined into a special cell at a temperature 420 °C and a pressure 30–40 GPa, according to the procedure proposed previously^79^. In order to obtain the uniform controlled oxygen content, the crystal after untwined was repeatedly annealed for seven days in an oxygen atmosphere at 420 °C^80^.

Rectangular crystals of about 1.7 × 1.2 × 0.2 mm were selected from the same batch to perform the resistivity measurements. The smallest parameter of the crystal corresponds to the c-axis. The experimental geometry was selected so that the transport current vector was parallel to the ab-plane. The four-point probe technique with stabilized measuring current of up to 10 mA was used to measure the ab-plane resistivity, ρ_ab_(T) [40, and references therein]. Silver epoxy contacts were glued to the extremities of the crystal in order to produce a uniform current distribution in the central region where voltage probes in the form of parallel stripes were placed. The procedure for making contacts was completed by adding silver wires with a diameter of 0.05 mm and a three-hour annealing at a temperature of 200 °C in an oxygen atmosphere. Contact resistances below 1 Ω were obtained. The temperature was measured using a Pt sensor having an accuracy of about 1 mK. The measurements were carried out in the temperature drift mode on two opposite directions of the transport current to eliminate the influence of the parasitic signal. The critical temperature, T_c_, was determined by extrapolation of the linear part of the SC transition to its intersection with the axis T^4,5,20,21^.

In order to change the oxygen content and, and obtain the appropriate values of n_f_ and T_c_, the sample was annealed for two days in an oxygen flow at temperature 620 °C. After annealing, the crystal was cooled to room temperature within 2–3 minutes, mounted in a measuring cell, and cooled to the temperature of liquid nitrogen for 10–15 minutes (sample A1). All measurements were carried out by heating the sample. To study the effect of annealing at room temperature, the sample after the first measurements of ρ(T) was kept for 20 hours at room temperature (sample A2) and then repeated measurements were performed. The following measurements were carried out after additional exposure of the sample at room temperature for three days (sample A3). After this procedure, not only increased T_c_ and decreased ρ(Т), but unlike the data of the previous work^7^, the PG temperature T* also decreased noticeably, whereas the value of PG increased, which is in full agreement with the PD for YBCO (refer to^3–5,13,21^ and references therein).
